# Tumor Variant Identification That Accounts for the Unique Molecular Landscape of Pediatric Malignancies

**DOI:** 10.1093/jncics/pky079

**Published:** 2019-01-25

**Authors:** Amanda Lorentzian, Jaclyn A Biegel, D Gigi Ostrow, Nina Rolf, Chi-Chao Liu, S Rod Rassekh, Rebecca J Deyell, Timothy Triche, Kirk R Schultz, Jacob Rozmus, Gregor S D Reid, C James Lim, Philipp F Lange, Christopher A Maxwell

**Affiliations:** 1 Department of Pediatrics, University of British Columbia, Vancouver, BC, Canada; 2 Department of Pathology and Laboratory Medicine, Children's Hospital Los Angeles; 3 Keck School of Medicine at University of Southern California, Los Angeles, CA; 4 Michael Cuccione Childhood Cancer Research Program, BC Children’s Hospital, Vancouver, BC, Canada; 5 Department of Pathology, University of British Columbia, Vancouver, BC, Canada

## Abstract

Precision oncology trials for pediatric cancers require rapid and accurate detection of genetic alterations. Tumor variant identification should interrogate the distinctive driver genes and more frequent copy number variants and gene fusions that are characteristics of pediatric tumors. Here, we evaluate tumor variant identification using whole genome sequencing (n = 12 samples) and two amplification-based next-generation sequencing assays (n = 28 samples), including one assay designed to rapidly assess common diagnostic, prognostic, and therapeutic biomarkers found in pediatric tumors. Variant identification by the three modalities was comparable when filtered for 151 pediatric driver genes. Across the 28 samples, the pediatric cancer-focused assay detected more tumor variants per sample (two-sided, *P *<* *.05), which improved the identification of potentially druggable events and matched pathway inhibitors. Overall, our data indicate that an assay designed to evaluate pediatric cancer-specific variants, including gene fusions, may improve the detection of target-agent pairs for precision oncology.

Pediatric cancers are thought to arise in developing tissues (1) and are commonly characterized by gene fusions and copy number variants (CNV) (2,3). Moreover, pediatric cancers have distinctive driver lesions and somatic mutation rates ranging from 10- to 100-fold lower than those in adult cancers (2,3). These genetic hallmarks of pediatric cancers should be integrated into next-generation sequencing (NGS) approaches to tumor variant identification for precision oncology.

Precision oncology has potential to improve treatment options for pediatric cancer patients but faces many obstacles, including the rapid identification of tumor variants. Whole genome sequencing (WGS) is the most comprehensive platform for cancer genome profiling but requires long turnaround times (4). Whole exome sequencing (WES) requires less time but is less able to detect gene fusions, which can be complemented by RNA sequencing but this integrative approach is less sensitive than WGS (4). Lastly, cancer genome profiling for specific molecular variants that serve as predictive biomarkers is a more rapid approach, which is used by the National Cancer Institute Molecular Analysis for Therapy Choice (MATCH) and Pediatric MATCH trials (5,6).

Target identification in the Pediatric MATCH trial (6) uses an amplification-based NGS assay known as Oncomine Comprehensive Assay version 3 (OCAV3), which requires less nucleic acid input than hybrid-capture assays and is compatible with degraded DNA and RNA isolated from formalin-fixed, paraffin-embedded (FFPE) tissues. However, OCAV3 was originally developed to screen informative variants for relapsed or refractory adult solid tumors or lymphomas (7), with the current version 3 including a set of pediatric-specific variants. We hypothesized that a pediatric-focused sequencing assay (8) designated the Oncomine Childhood Cancer Research Assay (OCCRA) will be more informative for variant detection in pediatric malignancies. To test this hypothesis, we retrospectively collected 28 childhood tumor samples from nine cancer types archived at diagnosis or relapse as frozen cells or FFPE tissue (Supplementary Table S1, available online). Briefly, we extracted DNA and RNA and performed the two NGS assays in parallel to identify CNVs, gene fusions, and single nucleotide variants (SNV) (Supplementary Methods, available online). Variants classified as benign or likely benign or variants of undetermined significance were filtered out (Supplementary Table S2, available online) but tended to function in cellular pathways associated with pediatric cancer predisposition genes (2), including DNA damage response (*ATM, SLX4, PALB2*), receptor tyrosine kinase signaling (*KIT, MET*), and epigenetic or transcription pathways (*SETD2, TET2*). For 12 samples, WGS data was available from matching samples previously sequenced by the Personalized Onco-Genomics program (9). All variants were winnowed against 151 pediatric cancer driver genes (Supplementary Table S3, available online) collated from recent WGS and WES datasets (2,3).

Both NGS assays identified at least one mutation in the majority of tumor samples (Figure 1A) (Supplementary Figure S1, available online). The pediatric-focused OCCRA detected more variants per sample (mean [SD] values = 2.1 [1.7] for OCCRA; 1.8 [1.7] for OCAV3) (Figure 1B), including a greater number of CNVs and gene fusions (Figure 1C). In 17 out of the 28 samples, the number of variants detected was identical between the assays although dissimilar variants were identified in two of these samples (Nos. 19 and 24) (Supplementary Figure S1, available online); in 8 of the remaining 11 samples, OCCRA detected more variants than OCAV3 (Figure 1A). The improved detection of gene fusions (*NUP214-ABL1*, *ETV6-RUNX1*, *STIL-TAL*) reflects OCCRA content, which contains roughly two times more RNA-based probes (Supplementary Tables S4 and S5, available online). In addition, OCCRA detected the loss of *CDKN2A* in more samples (Supplementary Figures S1 and S2, available online). *CDKN2A* is the most frequently mutated gene reported in pediatric cancers (3), and its loss was the most frequent variant observed in our analysis (Supplementary Figure S1, available online). Orthogonal clinical data and WGS-confirmed OCCRA detected nine of nine verifiable samples with *CDKN2A* loss compared to six of nine samples detected by OCAV3 (Supplementary Figure S2, available online). For a subset of samples, we evaluated the summarized WGS data from matched but not identical samples. The three NGS methodologies detected similar mean variants per sample (Figure 1B) with at least one mutation detected in most samples (Supplementary Figure S1, available online). SNV detection was near identical between the modalities (Figure 1D); however, CNV detection was less similar, which likely reflects both the quality and the biology of the samples. For instance, tumor content in sample 18 was twofold lower than the matched sample that was previously evaluated by WGS (23% vs 40%). Removal of sample 18 greatly improves the uniformity of CNV detection (Figure 1D). Samples 20 and 27 contain extensive aneuploidy by WGS and widespread gains and losses that diverge depending on panel content (Supplementary Tables S1 and S2, available online). Divergent variant detection in these samples may indicate considerable rearrangement of chromosomes or chromothripsis, which occurs in 11% of pediatric cancer samples (3). Overall, our detection of 1–3 variants per sample, with CNVs accounting for the majority of variants, is comparable to recent WGS and WES pan-cancer datasets (2,3).

**Figure 1.**
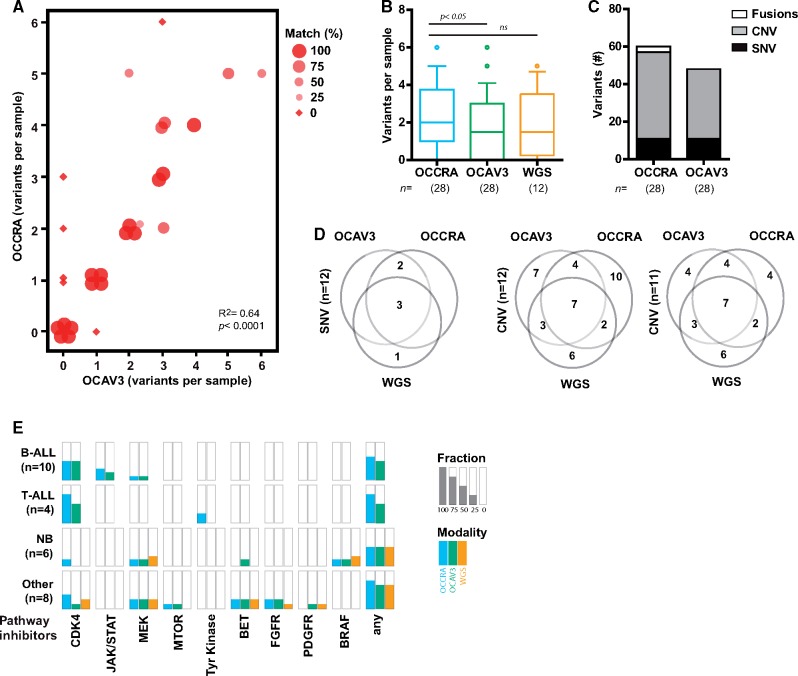
Improved variant discovery using childhood cancer-specific amplicon-based sequencing. **A**) Variant discovery across 28 tumor samples using Oncomine Childhood Cancer Research Assay (OCCRA) and Oncomine Comprehensive Assay Version 3 (OCAV3) next-generation sequencing assays. The size and shape of the marker indicates the percentage of concordance (identical variants/total variants) between the assays. **B**) OCCRA detected more variants per sample than OCAV3 (n = 28, paired *t*-test, two tail). For the 12 samples analyzed by three modalities, the variants detected per sample were not statistically different (n = 12, one-way ANOVA with Tukey multiple comparison). Data plotted as box and whiskers (10–90 percentile). **C**) The types of variant detected included more copy number variants (CNV) and fusions in samples assayed by OCCRA than parallel analyses with OCAV3. **D**) Venn diagrams for variants in pediatric cancer driver genes detected by each assay compared to whole genome sequencing (WGS) shows high concordance in the detection of single nucleotide variants (SNV) but less for CNVs, which is improved by the removal of sample No. 18 (20% tumor content). **E**) The fraction of samples that were matched with the indicated pathway inhibitors for each modality across the indicated tumor types.

We assigned target-agent pairs to each sample using the Pediatric MATCH strategy (6) and evidence from clinical trials or case reports (Supplementary Methods, available online). The pediatric-focused assay detected at least one target-agent pair for 18 of the 28 samples (64%) (Figure 1E). At least one target-agent pair was detected for 15 of the 28 samples sequenced by OCAV3 (54%) and 7 of 12 samples by WGS (58%) (Figure 1D), which are comparable to the frequency of potentially druggable events identified by WGS and WES in childhood cancers (52%) (2).

Our analysis is limited to a small, single-site cohort and should be confirmed in a multicenter study. Moreover, amplification-based sequencing will not detect common IGH, IGK, or IGL rearrangements as it requires PCR primers against both partners in a fusion (8). Finally, caution is merited as rational target-agent pairs may prove ineffective in practice. For example, *CDKN2A* mutant cells show only intermediate sensitivity to CDK4/6 inhibition due to compensatory phosphorylation by CDK2 (10). Such molecular compensation may be revealed through integration of proteomic pathway analysis with genome profiling. In conclusion, the detection of variants in pediatric driver genes was comparable between WGS and a rapid, pediatric cancer-focused NGS assay. Variant detection using this assay was superior to an adult cancer-focused assay and, thus, may better enable precision oncology clinical trials for molecularly guided therapies in childhood cancer patients.

## Supplementary Material

Supplementary Data
