# Supplementary material for: Tumor Variant Identification That Accounts for the Unique Molecular Landscape of Pediatric Malignancies
Source: JNCI Cancer Spectr. 2019 Jan 25;2(4):pky079. doi: 10.1093/jncics/pky079 (PMC6447067; doi:10.1093/jncics/pky079)
Supplement: Supplementary Data [file pky079_supp.pdf]

Supplementary Materials

Tumor variant identification that accounts for the unique molecular landscape of pediatric malignancies

Supplemental Methods

Tumour samples. All tumour samples were retrospectively sourced from the Biobank at BC Children's Hospital (BCCH) following approval by the University of British Columbia Children's and Women's Research Ethics Board (REB #H17-01860).

Amplicon-based sequencing and variant determination. Extraction of DNA (RecoverAll, Thermo Fisher Scientific (TFS)) and RNA (Allprep, Qiagen), library preparation, and targeted sequencing on the Ion Chef and Ion Torrent S5 platforms followed manufacturer's protocols (TFS). Oncomine Comprehensive Assay version 3 (OCAV3) includes 2,290 unique DNA-based amplicons to detect SNVs and CNVs as well as 867 RNA-based amplicons to detect unique fusions or structural variants. Oncomine Childhood Cancer Research Assay (OCCRA) includes 2,031 unique DNA-based and 1,701 RNA-based amplicons. Detection sensitivities include: hotspot mutations (OCAV3: 99.2%; OCCRA: 99%), Indels (OCAV3: 96.9%; OCCRA: 100%), and fusions (OCAV3: 95.4%; OCCRA: 92.2% or 82.9% for blood or tissue samples) [1, 2]. Average read depth for DNA and RNA for both panels was approximately $9x10^6$-$12x10^6$ and $8x10^5$ - 1 $x10^6$, respectively.

SNVs, including those in pediatric cancer driver genes, non-driver genes, variants of undetermined significance and benign/likely benign variants, were retrieved with Ion Reporter software (version 5.2). Copy number (CN) measurements were retrieved with Ion Reporter software (version 5.2) for genes with >5 probes, including those that were validated for CN gains (Table S5). We noted frequent detection of homogenous loss for CDKN2A, which is not validated in either panel. To determine cut-offs for CN loss and CN gain that were verifiable by orthogonal clinical reports and/or whole genome sequencing, CN measurements for 14 genes captured by both panels were plotted and true positive calls for CN gains or losses were marked (Figure S2). A cut-off for loss at CN <1.1 and a cut-off for gain at CN >3.5 gave 35 abnormal CN calls in these 14 genes; 19 of those calls (7 gains, 12 losses) were verifiable by clinical reports and/or whole genome sequencing. For these 19 true positive calls, the OCCRA panel detected 18 of 19 (95%)(one false negative: sample 27 gave PDGFRA at CN=3) while OCAV3 panel detected 16 of 19 (80%)(three false negatives: sample 13 gave CDKN2A at CN> 1.1; sample 16 gave MYCN at CN> 1.1; sample 23 gave CDKN2AB at CN> 1.1)(Figure S2).

Archived and summarized whole genome sequencing data from the analysis of matched samples, when available, was provided by the Personalized Onco-Genomics program [3].

Data winnowing for pediatric cancer driver genes. Variants that were detected and filtered out of the analysis are tabulated in Table S2. These variants were filtered out when: (1) the variation occurred in genes not

considered to be driver genes for pediatric tumors (indicated by a *, Table S2); (2) the variant is of undetermined significance (indicated by a ǂ, Table S2); or, (3) the variant is benign/likely benign (indicated by a ^, Table S2).

Variant – agent determination. Variant-agent pairs were determined using the Pediatric MATCH prioritization strategy [4]. Variant- agent pairs supported by clinical trials or case reports, including JAK1 variants with JAK/STAT inhibitors [5] and NUP214-ABL1 with tyrosine kinase inhibitors [6, 7], were also included.

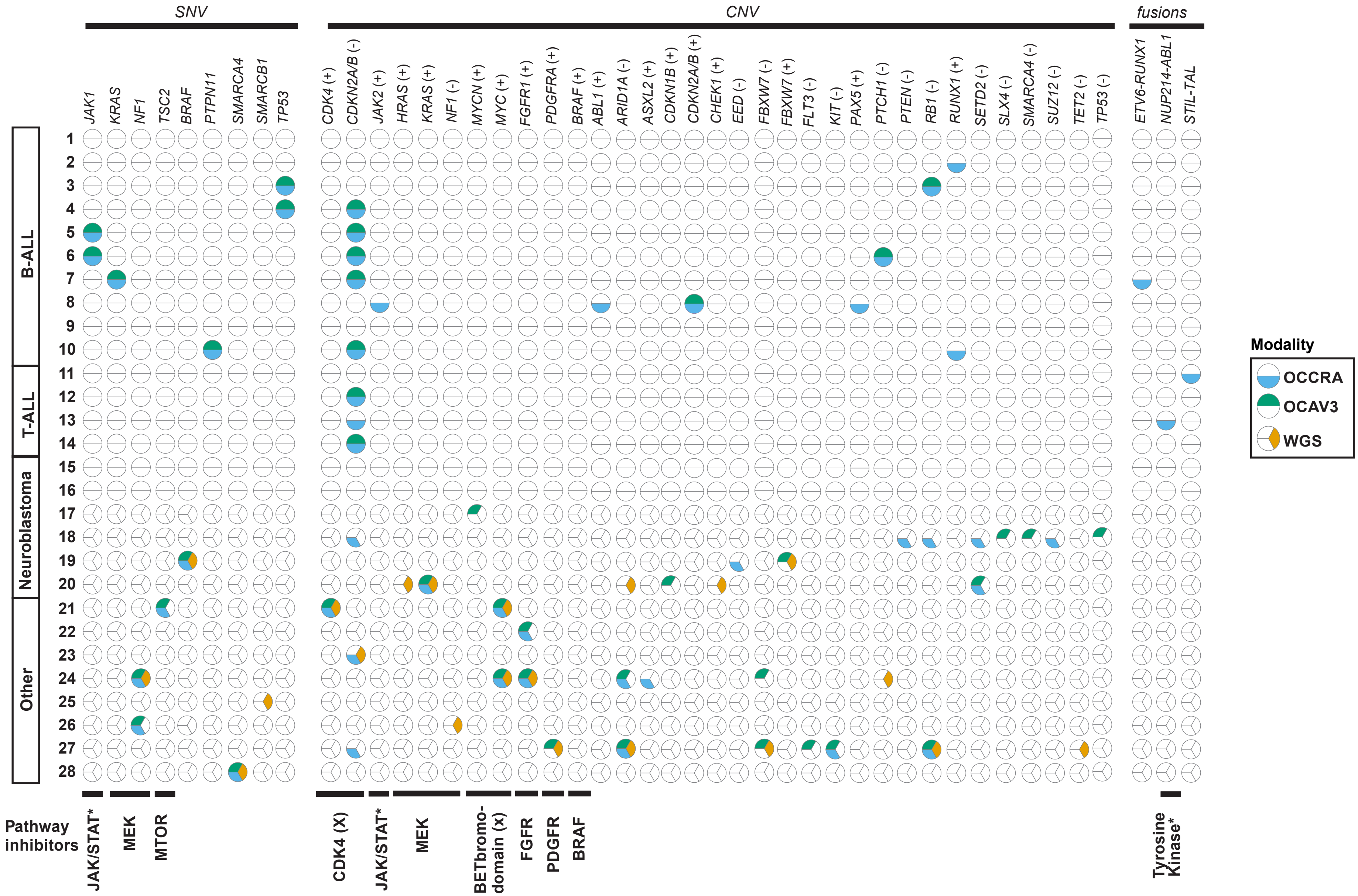

Figure S1. Comparison between whole genome or amplicon-based sequencing detection of pediatric cancer driver genes.
Single nucleotide variants (SNV), copy number variants (CNV) and fusions were assayed across 28 samples using amplicon-based sequencing and, for the final 12 samples, whole genome sequencing (WGS). The detection of a variant is indicated by a filled half- or semi-circle with the color corresponding to the modality that detected the variant. Using the strategy outlined by the Pediatric MATCH target-agent prioritization committee, target - inhibitor pairs were determined for each sample. Inhibitors to those pathways that were reviewed by the committee but are not currently included in Pediatric MATCH are designated with an (X). Agents that were not included for review by Pediatric MATCH are indicated by an asterisk.

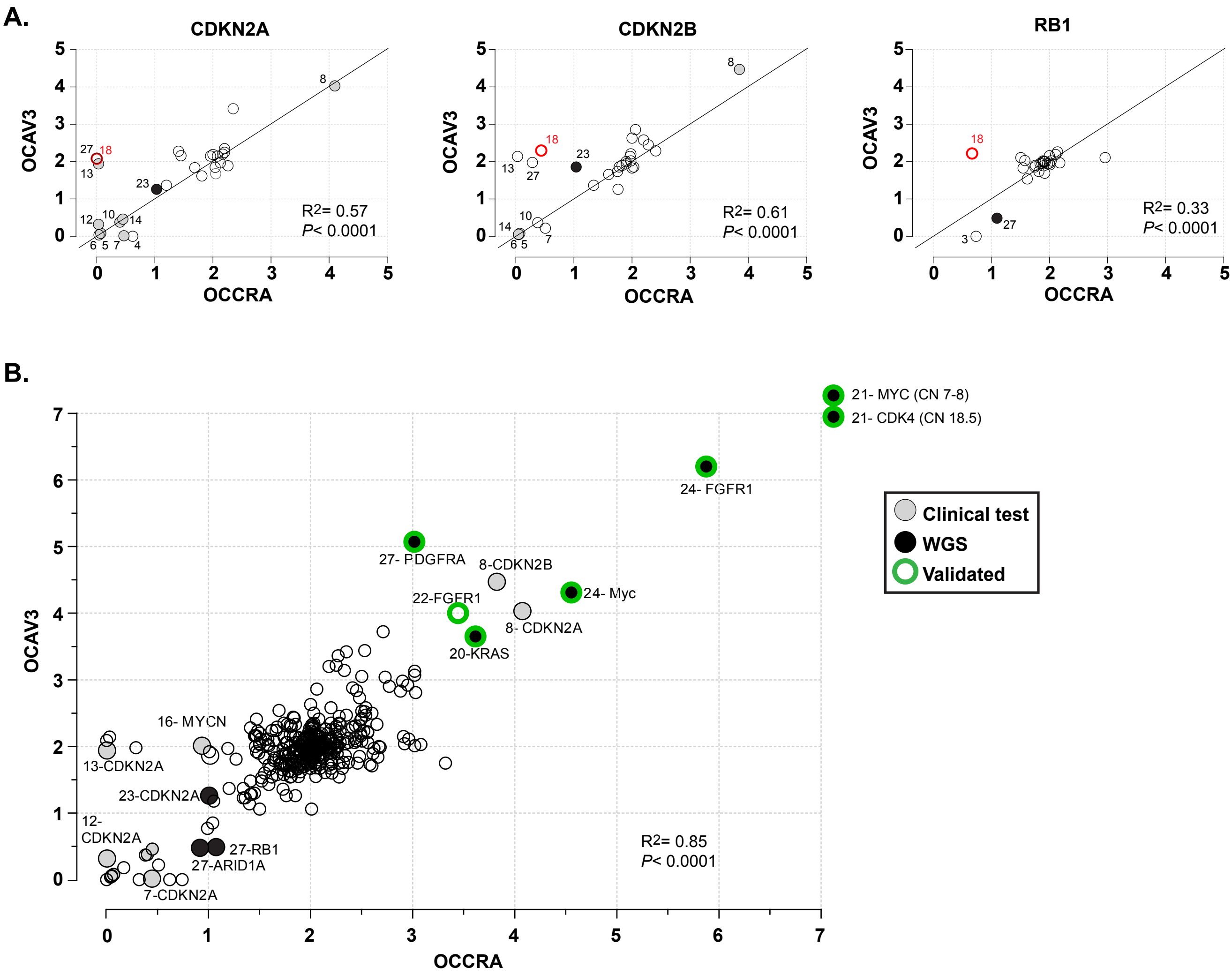

Figure S2. Congruent detection of copy number variation by amplicon-based sequencing.

A. Copy number measurements for indicated genes across 28 samples. Sample #18 (red), which had low tumor content (<20%), gave discordant measurements. Selected gene-sample measurements are highlighted as follows: gains/ losses that were also observed by whole genome sequencing (WGS) are indicated by black circles; gains/ losses that were annotated in clinical reports are indicated by grey circles.

B. Copy number measurements for those genes that are common between OCCRA and OCAV3 and contained >5 probes across 27 samples (n=378 measurements per panel). Data obtained from sample #18 is excluded. Selected gene-sample measurements are highlighted as follows: gene probes that were validated for amplification are circled in green; gains/ losses that were also observed by whole genome sequencing (WGS) are indicated by black circles; gains/ losses that were annotated in clinical reports are indicated by grey circles.

Supplemental Tables

Table S1: SNVs, CNVs, and fusions, filtered for pediatric cancer driver genes, are tabulated for amplicon-based (OCCRA or OCAV3) and whole genome sequencing (WGS). Clinical data was extracted for samples 1 – 16. Not available, N/A.

			**Tumor content**		**SNVs** (allele frequency)			**CNVs** (copy number)			**Fusions** (counts)	
**Sample**	**Patient**	**Cancer Type**	**WGS**	**Panels**	**OCCRA**	**OCAv3**	**WGS**	**OCCRA**	**OCAv3**	**WGS**	**OCCRA**	**OCAv3**
1	1	B-ALL	-	96	0	0	-	0	0	-	0	0
2	2	B-ALL	-	79	0	0	-	**RUNX1** (4.35)	0	-	0	0
3	3-Diagnosis	B-ALL	-	83	**TP53** (0.82) Arg248Gln	**TP53** (0.79) Arg248Gln	-	**RB1** (0.74) [^]	**RB1** (0) [^]	-	0	0
4	3-Relapse2	B-ALL	-	67	**TP53** (0.80) Arg248Gln	**TP53** (0.82) Arg248Gln	-	**CDKN2A** (0.62) [^]	**CDKN2A** (0) [^]	-	0	0
5	4-Relapse2	B-ALL	-	96	**JAK1** (0.48) Val658Phe	**JAK1** (0.46) Val658Phe	-	**CDKN2A** (0.07) [ǂ,^]<br>**CDKN2B** (0.07) [^]	**CDKN2A** (0.08) [ǂ,^]<br>**CDKN2B** (0.08) [^]	-	0	0
6	4-Relapse5	B-ALL	-	90	**JAK1** (0.98) Val658Phe	**JAK1** (0.98) Val658Phe	-	**CDKN2A** (0.04) [^]<br>**CDKN2B** (0.04) [^]<br>**PTCH1** (1.0) [^]	**CDKN2A** (0.05) [^]<br>**CDKN2B** (0.05) [^]<br>**PTCH1** (0.9) [^]	-	0	0
7	5	B-ALL	-	92	**KRAS** (0.16) Gly12Ser	**KRAS** (0.19) Gly12Ser	-	**CDKN2A**(0.47) [ǂ,^]<br>**CDKN2B** (0.51) [^]	**CDKN2A**(0.17) [ǂ,^]<br>**CDKN2B** (0.22) [^]	-	**ETV6-RUNX1** (922510)	0
8	6-Relapse1	B-ALL	-	N/A	0	0	-	**JAK2** (4.00) [ǂ]<br>**CDKN2A**(4.10) [ǂ,^]<br>**CDKN2B** (3.9) [ǂ,^]<br>**PAX5**(4.00) [ǂ]<br>**ABL1** [ǂ] (3.54)	**CDKN2A**(4.03) [ǂ,^]<br>**CDKN2B** (4.47) [ǂ,^]	-	0	0
9	7	B-ALL	-	97	0	0	-	0	0	-	0	0
10	8	B-ALL	-	90	**PTPN11**(0.37) Glu76Lys	**PTPN11**(0.38) Glu76Lys	-	**CDKN2A**(0.40) [ǂ,^]<br>**CDKN2B** (0.38) [^]<br>**RUNX1** (3.71)	**CDKN2A**(0.38) [ǂ,^]<br>**CDKN2B** (0.37) [^]	-	0	0
11	9	T-ALL	-	90	0	0	-	0	0	-	**STIL-TAL** (5678)	0
12	10	T-ALL	-	81	0	0	-	**CDKN2A**(0.03) [ǂ,^]	**CDKN2A**(0.32) [ǂ,^]	-	0	0
13	11	T-ALL	-	96	0	0	-	**CDKN2A** [ǂ] (0.03)<br>**CDKN2B** (0.03)	0	-	**NUP214-ABL1** (12524)	0
14	12	T-ALL	-	83	0	0	-	**CDKN2A** [ǂ,^] (0.45)<br>**CDKN2B** [ǂ,^] (0.05)	**CDKN2A** [ǂ,^] (0.46)<br>**CDKN2B** [ǂ,^] (0.06)	-	0	0
15	13	Neuroblastoma	-	N/A	0	0	-	0	0	-	0	0
16	14	Neuroblastoma	-	N/A	0	0	-	0	0	-	0	0
17	15	Neuroblastoma	80%	N/A	0	0	0	0	**MYCN** [*] (3.7)	0	0	0
18	16	Neuroblastoma	41%	23%	0	0	0	**CDKN2A** (0)<br>**CDKN2B** (0.44)<br>**PTEN** (0.53)<br>**RB1** (0.67)<br>**SETD2** (0.67)<br>**SUZ12** (0.53)	**SLX4** (0.84)<br>**TP53**(0.53)<br>SMARCA4 (1.07)	0	0	0

19	17-Relapse5	Neuroblastoma	90%	58%	**BRAF** (0.54) Gly469Ala	**BRAF** (0.56) Gly469Ala	**BRAF** Gly469Ala	**EED** (0.45)	**FBXW7**[^] (3.5)	**FBXW7**[^] (gain)	0	0
20	18-Relapse1	Neuroblastoma	45%	43%	0	0	0	**SETD2**[^] (0.33)<br>**KRAS**[*,^] (3.7)	**SETD2**[^] (0)<br>**CDKN1B** (3.57)<br>**KRAS**[*,^] (3.7)	**CHEK1**(Gain)<br>**KRAS** (Gain)<br>**HRAS** (Gain)<br>**ARID1A**(Loss)	0	0
21	19	Osteosarcoma	78%	95%	**TSC2** (0.63) Tyr190Ter	**TSC2** (0.67) Tyr190Ter	0	**CDK4**[*,^] (15.3)<br>**MYC**[*,^] (7.16)	**CDK4**[*,^] (18.68)<br>**MYC**[*,^] (8.27)	**CDK4**[*,^]<br>**MYC**[*,^]	0	0
22	20	Astrocytoma	41%	80%	0	0	0	**FGFR1**[*,^] (3.5)	**FGFR1**[*,^] (4.0)	0	0	0
23	21	Glioblastoma	58%	20%	0	0	0	**CDKN2A**[^] (1.0)<br>**CDKN2B**[^] (1.0)	0	**CDKN2A**(loss)[^]<br>**CDKN2B**(loss)[^]	0	0
24	22-Relapse1	Rhabdo-myosarcoma	98%	70%	**NF1** (0.44) Glu318fs	**NF1** (0.44) Glu318fs	**NF1** Glu318Lysfs	**ARID1A**[^] (0.17)<br>**FGFR1**[*,^] (5.91)<br>**MYC**[^] (4.59)<br>**ASXL2** (3.65)	**ARID1A**[^] (0.19)<br>**FBXW7** (0.33)<br>**FGFR1**[*,^] (6.29)<br>**MYC**[3] (4.31)	**FGFR1**[*,^] (Gain)<br>**MYC**[^] (Gain)<br>**PTCH1** (Loss)	0	0
25	23	Rhabdoid (kidney)	23%	43%	0	0	**SMARCB1** Glu300Aspfs	0	0	0	0	0
26	24-Relapse1	Neurofibroma	35%	60%	**NF1** (0.59) Arg2637Ter	**NF1** (0.55) Arg2637Ter	0	0	0	**NF1** (loss)	0	0
27	25	Glioblastoma	88%	63%	0	0	0	**ARID1A**[^] (0.95)<br>**CDKN2A** (0)<br>**CDKN2B** (0.29)<br>**KIT**[^] (0.99)<br>**RB1**[^] (1.10)	**ARID1A**[^] (0.48)<br>**FBXW7**[^] (0.64)<br>**FLT3** (0.88)<br>**KIT**[^] (0.77)<br>**PDGFRA**[*,^] (5.07)<br>**RB1**[^] (0.50)	**ARID1A**[^] (loss)<br>**FBXW7**[^] (loss)<br>**PDGFRA**[^] (gain)<br>**RB1**[^] (loss)<br>**TET2** (loss)	0	0
28	26	Rhabdoid (kidney)	76%	65%	**SMARCA4**(0.38) Gln144Ter	**SMARCA4**(0.39) Gln144Ter	**SMARCA4** Gln144Ter	0	0	0	0	0

* variants detected with probes validated for CNV

ǂ variants confirmed by clinical tests

^ variants detected by >1 modality

Table S2: Summary of filtered out SNVs, CNVs, and fusions for amplicon-based (OCCRA or OCAV3) and whole genome sequencing (WGS). Not available, N/A.

Sample	Patient	Cancer Type	**Tumor content**		**SNVs** (allele frequency)			**CNVs** (copy number)			**Fusions** (counts)	
			**WGS**	**Panels**	**OCCRA**	**OCAv3**	**WGS**	**OCCRA**	**OCAv3**	**WGS**	**OCCRA**	**OCAv3**
1	1	B-ALL	-	96	0	**ATM**^ (0.48)<br>Asp1853Asn	N/A	0	0	-	0	0
2	2	B-ALL	-	79	0	**ATM**^ (0.49)<br>Asp1853Asn	N/A	0	0	-	0	0
3	3-<br>Diagnosis	B-ALL	-	83	0	0	N/A	0	0	-	0	0
4	3-<br>Relapse2	B-ALL	-	67	0	0	N/A	0	0	-	0	0
5	4-<br>Relapse2	B-ALL	-	96	0	**SETD2**‡ (0.49)<br>Tyr1481Ter	N/A	0	0	-	0	0
6	4-<br>Relapse5	B-ALL	-	90	0	**SETD2**‡ (0.53)<br>Tyr1481Ter	N/A	0	0	-	0	0
7	5	B-ALL	-	92	0	0	N/A	0	0	-	0	0
8	6-<br>Relapse1	B-ALL	-	N/A	0	0	N/A	0	0	-	0	0
9	7	B-ALL	-	97	0	0	N/A	0	0	-	0	0
10	8	B-ALL	-	90	0	**ATM**^ (0.46)<br>Asp1853Asn	N/A	0	0	-	0	0
11	9	T-ALL	-	90	0	0	N/A	0	0	-	0	0
12	10	T-ALL	-	81	**FBXW7**^(0.04)<br>Arg465His	**FBXW7**^(0.04<br>Arg465His	N/A	0	0	-	0	0
13	11	T-ALL	-	96	0	**SLX4**‡ (0.53)<br>Gln1632fs	N/A	**CRLF1*** (0.93)	0	-	0	0
14	12	T-ALL	-	83	0	0	N/A	0	0	-	0	0
15	13	Neuroblastoma	-	N/A	0	0	N/A	0	0	-	0	0
16	14	Neuroblastoma	-	N/A	0	**KIT**^ (0.49)<br>Met541Leu	N/A	**ABL2*** (4.01)<br>**MYCN**‡ (0.96)	0	-	0	0
17	15	Neuroblastoma	80%	N/A	0	0	0	**ERBB2*** (4.86)	**CDK12*** (4.0)<br>**ERBB2*** (4.32)<br>**RNF43*** (4.14)<br>**RAD51C*** (4.04)	0	0	0
18	16	Neuroblastoma	41%	23%	0	**SLX4**‡ (0.53)<br>Gln1632fs	0	**KRAS**‡ (1.02)<br>**MYCN**‡ (0.93)	0	0	0	0
19	17-<br>Relapse5	Neuroblastoma	90%	58%	0	**ATM*** (0.79)<br>Arg337His	**PIK3CA**‡<br>Gly12Asp<br>**NOTCH1**‡<br>Thr194Pro	**CCND1*** (11.60)	**CCND1*** (9.47)<br>**FGF3*** (8.38)<br>**FGF19*** (8.56)	**CCND1*** (gain)<br>**AURKA*** (gain)<br>**SMAD4*** (gain)<br>**ERBB2*** (gain)<br>**LRP1B*** (gain)<br>**CDK6*** (gain)<br>**INPP4B*** (gain)	0	0
20	18-<br>Relapse1	Neuroblastoma	45%	43%	0	**MET*** (0.51)<br>Glu168Asp	**ROS1**‡<br>Ile1530Thr	**CCND1*** (7.76)	**CCND1*** (6.47)<br>**FGF3*** (6.02)	**SFPQ*** (loss)<br>**MLH1*** (loss)<br>**CLSPN*** (loss)	0	0

										**XPC**[*] (loss)<br>**CUL5**[*] (loss)<br>**FANCD2**[*] (loss)<br>**ATM**[*] (loss)<br>**MUTYH**[*] (loss)<br>**CCND1/2**[*] (gain)<br>**FGF3/4**[*] (gain)<br>**ETV4**[*] (gain)<br>**GNAS**[*] (gain)<br>**NTRK1**[*] (gain)<br>**MDM4**[*] (gain)		
21	19	Osteosarcoma	78%	95%	0	0	0	**GLI1**[*] (15.3)	0	**CCNE1**[*] (gain)<br>**DMD**[*] (loss)<br>**LSAMP**[*] (loss)	0	0
22	20	Astrocytoma	41%	80%	0	0	0	**ABL2**[*] (5.35)<br>**FASLG**[*] (5.29)<br>**MDM4**[*] (5.21)	**DDR2** [*] (5.39)<br>**MDM4**[*] (4.14)<br>**NTRK1**[*] (4.16)	**MCL1**[*] (gain)<br>**MDM4**[*] (gain)	0	0
23	21	Glioblastoma	58%	20%	0	0	0	0	0	0	0	0
24	22-<br>Relapse1	Rhabdo-<br>myosarcoma	98%	70%	**PPM1D**[*]<br>(0.55)<br>Glu525Ter	0	**POLQ**[*]<br>Q2556Hfs	**AKT1**[*] (0.87)<br>**ARID1B**[*] (0.24)<br>**CHD7**[*] (5.71)<br>**MTOR**[*] **(**0.84)<br>**KRAS**[ǂ] (1.05)	**NBN**[*] (5.47)<br>**RAD51B**[*] (0.86)<br>**KRAS**[ǂ] (0.9)	0	0	0
25	23	Rhabdoid<br>(kidney)	23%	43%	0	0	**SMARCB1**[ǂ]<br>Glu300Aspfs	0	0	0	0	0
26	24-<br>Relapse1	Neurofibroma	35%	60%	0	0	**PRSS3** [*,ǂ]<br>Arg255Gly<br>**ACSM5**[*,ǂ]<br>Asp400Asn	0	0	0	0	0
27	25	Glioblastoma	88%	63%	0	**PALB2**[ǂ] (0.43)<br>Arg170fs	0	0	0	**FUBP1**[*] (loss)<br>**FAT1**[*] (loss)<br>**SDHB**[*] (loss)<br>**CDKN2C**[*] (loss)<br>**ERCC5**[*] (loss)<br>**ING1**[*] (loss)<br>**NFKB1**[*] (loss)<br>**MUTYH**[*] (loss)<br>**BRCA2**[*] (loss)<br>**ATK3**[*] (gain)<br>**ABL2**[*] (gain)	0	0
28	26	Rhabdoid<br>(kidney)	76%	65%	**TET2**[*] (0.45)<br>Val1718Leu	0	0	0	0	0	0	0

* variants in genes that are not frequently mutated in pediatric cancers

ǂ variants of undetermined significance

^ benign/likely benign variants

Table S3: 151 pediatric driver genes. List derived from 77 significantly mutated genes [8] and the top 100 recurrently mutated genes in pediatric tumors [9] cross-referenced to remove duplicates.

AADACL4	CBFB	EP300	JAK2	NIPBL	PTPRD	STAG2	WDR64
ABL1	CBL	ERG	JAK3	NKX2-1	RAG1	STAT5B	WHSC1
ACVR1	CCND3	ETV6	KBTBD4	NOCR1	RB1	SUFU	WT1
ADD3	CDKN1B	FAT2	KDM68	NOTCH1	RHOA	SUZ12	WZH2
ALK	CDKN2A	FBN2	KDM6A	NOTCH2	ROS1	TAL1	XBP1
ARID1A	CDKN2B	FBXO11	KIT	NPM1	RPL10	TBL1RX1	ZEB2
ARID2	CDK4	FBXW7	KMT2A	NRAS	RPL22	TBR1	ZFP36L2
ASXL	CEBPA	FGFR1	KMT2C	NUP98	RUNX1	TCF3	ZIC1
ASXL2	CHD4	FLG	KMT2D	P2RY8	SELP	TCF7	ZMYM3
ATF7IP	COL1A1	FLT3	KRAS	PAX5	SETD2	TERT	ZNF217
ATRX	CREBBP	FMR1	LAPTM4B	PCBP1	SF3B1	TET2	ZNF384
BCL11B	CRLF2	GATA2	LEF1	PDGFRA	SH2B3	TFAP4	
BCOR	CTCF	H3F3A	LMO2	PHF6	SHANK2	TLX1	
BCORL1	CTNNB1	HDAC2	MED12	PIK3CA	SI	TLX3	
BCR	DDX3X	HIST1H3B	MGA	PIK3R1	SIRPA	TOX	
BPIFB1	DGCR8	ID3	MLLT10	PTCH1	SIX1	TP53	
BRAF	DNM2	IDH1	MYB	PTCHD4	SMARCA4	UBA2	
BTG1	DROSHA	IKZF1	MYC	PTEN	SMARCB1	USP7	
C10orf112	EBF1	IL7R	MYCN	PTPN11	SMG8	USP9X	
CARD11	EED	JAK1	NF1	PTPN2	SMO	WAC	

Table S4: Genes and fusions covered by OCAV3 and OCCRA sequencing panels

OCCRA							OCAV3				
Hotspot Genes		Full Exon	CNV	Fusion		Expression	Hotspot Genes		Full Genes	CNV	Fusions
ABL1	ABL2	APC	ALK	ABL1	ABL2	BCL2	AKT1	AKT2	ATM	AKT1	ALK
ALK	ACVR1	ARID1A	BRAF	ALK	BCL11B	BCL6	AKT3	ALK	BAP1	AR	AXL
AKT1	ASXL1	ARID1B	CCND1	BCOR	BCR	FGFR1	AR	ARAF	BRCA1	CCND1	BRAF
ASXL2	BRAF	ATRX	CDK4	BRAF	CAMTA1	FGFR4	AXL	BRAF	BRCA2	CCNE1	EGFR
CALR	CBL	CDKN2A	CDK6	CCND1	CIC	IGF1R	BTK	CBL	CDKN2A	CDK4	ERBB2
CCND3	CCR5	CDKN2B	EGFR	CREBBP	CRLF2	MET	CCND1	CDK4	FBXW7	CDK6	ERG
CDK4	CIC	CEBPA	ERBB2	CSF1R	DUSP22	MYCN	CDK6	CHEK2	MSH2	EGFR	ETV1
CREBBP	CRLF2	CHD7	ERBB3	EGFR	ETV6	MYC	CSF1R	CTNNB1	NF1	ERBB2	ETV4
CSF1R	CSF3R	CRLF1	FGFR1	EWSR1	FGFR1	TOP2A	DDR2	EGFR	NF2	FGFR1	ETV5
CTNNB1	DAXX	DDX3X	FGFR2	FGFR2	FGFR3		ERB83	ERBB2	NOTCH1	FGFR2	FGFR1
DNMT3A	EGFR	DICER1	FGFR3	FLT3	FOSB		ERBB4	ERCC2	PIK3R1	FGFR3	FGFR2
EP300	ERBB2	EBF1	FGFR4	FUS	GLI1		ESR1	EZH2	PTCH1	FGFR4	FGFR3
ERBB3	ERBB4	EED	GLI1	GLIS2	HMGA2		FGFR1	FGFR2	PTEN	FLT3	NTRK1
ESR1	EZH2	FAS	GLI2	JAK2	KAT6A		FGFR3	FGFR4	RB1	IGF1R	NTRK3
FBXW7	FGFR2	GATA1	IGF1R	KMT2A	KMT2B		FLT3	FOXL2	SMARCB1	KIT	PDGFRA
FGFR3	FLT3	GATA3	KIT	KMT2C	KMT2D		GATA2	GNA11	STK11	KRAS	PPARG
GATA2	GNA11	GNA13	KRAS	LMO2	MAML2		GNAQ	GNAS	TP53	MDM2	RAF1
GNAQ	H3F3A	ID3	MDM2	MAN2B1	MECOM		H3F3A	HIST1H3B	TSC1	MDM4	RET
HDAC9	HIST1H3B	IKZF1	MDM4	MEF2B	MET		HNF1A	HRAS	TSC2	MET	ROS1
HRAS	IDH1	KDM6A	MET	MKL1	MLLT10		IDH1	IDH2	ARID1A	MYC	AKT2
IDH2	IL7R	KMT2D	MYC	MN1	MYB		JAK1	JAK2	ATR	MYCL	AR
JAK1	JAK2	MYOD1	MYCN	MYBL1	MYH11		JAK3	KDR	ATRX	MYCN	BRCA1
JAK3	KDM4C	NF1	PDGFRA	MYH9	NCOA2		KIT	KNSTRN	CDK12	PDGFRA	BRCA2
KDR	KIT	NF2	PIK3CA	NCOR1	NOTCH1		KRAS	MAGOH	CDKN1B	PIK3CA	CDKN2A
KRAS	MAP2K1	PHF6		NOTCH2	NOTCH4		MAP2K1	MAP2K2	CDKN2B	PPARG	ERB84
MAP2K2	MET	PRPS1		NPM1	NR4A3		MAP2K4	MAPK1	CHEK1	TERT	ESR1
MPL	MSH6	PSMB5		NTRK1	NTRK2		MAX	MDM4	CREBBP	AKT2	FGR
MTOR	NCOR2	PTCH1		NTRK3	NUP214		MED12	MET	FANCA	AKT3	FLT3
NOTCH1	NPM1	PTEN		NUP98	NUTM1		MTOR	MYC	FANCD2	ALK	JAK2
NRAS	NT5C2	RB1		NUTM2B	PAX3		MYCN	MYD88	FANCI	AXL	KRAS
PAX5	PDGFRA	RUNX1		PAX5	PAX7		NFE2L2	NRAS	MLH1	BRAF	MDM4
PDGFRB	PIK3CA	SMARCA4		PDGFB	PDGFRA		NTRK1	NTRK2	MRE11A	CCND2	MET
PIK3R1	PPM1D	SMARCB1		PDGFRB	PLAG1		PDGFRA	PDGFRB	MSH6	CCND3	MYB
PTPN11	RAF1	SOCS2		RAF1	RANBP17		PIK3CA	PIK3CB	NBN	CDK2	MYBL1
RET	RHOA	SUFU		RARA	RECK		PPP2R1A	PTPN11	NOTCH2	CDKN2A	NF1
SETBP1	SETD2	SUZ12		RELA	RET		RAC1	RAF1	NOTCH3	CDKN2B	NOTCH1
SH2B3	SH2D1A	TCF3		ROS1	RUNX1		RET	RHEB	PALB2	ESR1	NOTCH4
SMO	STAT3	TET2		SS18	SSBP2		RHOA	ROS1	PMS2	FGF19	NRG1
STAT5B	TERT	TP53		STAG2	STAT6		SF3B1	SMAD4	POLE	FGF3	NTRK2
TPMT	USP7	TSC1		TAL1	TCF3		SMO	SPOP	RAD50	NTRK1	NUTM1
ZMYM3		TSC2		TFE3	TP63		SRC	STAT3	RAD51	NTRK2	PDGFRB
		WHSC1		TSLP	TSPAN4		TERT	TOP1	RAD51B	NTRK3	PIK3CA
		WT1		UBTF	USP6		U2AF1	XPO1	RAD51C	PDGFRB	PRKACA
		XIAP		WHSC1	YAP1				RAD51D	PIK3CB	PRKACB
				ZMYND11	ZNF384				RNF43	RICTOR	PTEN
									SETD2	TSC1	RAD51B
									SLX4	TSC2	RB1
									SMARCA4		RELA
											RSPO2
											RSPO3
											TERT

Table S5: Genes with >5 probes filtered by pediatric cancer driving genes. Copy number (CN) results for the14 highlighted genes were used to optimize the definition of CN loss (<1.1) and CN gain (>3.5). Genes validated by the manufacturer to detect copy number gain indicated by an asterisk.

**OCCRA**			**OCAV3**		
ABL1	ACVR1	ALK*	ALK*	**ARID1A**	BRAF*
**ARID1A**	ASXL2	BRAF*	CCND3*	**CDK4***	CDKN1B
CBL	**CDK4***	**CDKN2A**	**CDKN2A***	**CDKN2B***	EP300
**CDKN2B**	CEBPA	CREBBP	FBXW7	**FGFR1***	FLT3*
CTNNB1	EBF1	EED	**KIT***	**KRAS***	**MYC***
**FGFR1***	GATA2	IKZF1	**MYCN***	NF1	NOTCH1
JAK1*	JAK2*	JAK3*	NOTCH2	**PDGFRA***	PIK3CA*
**KIT***	KMT2D	**KRAS***	PIK3R1	PTCH1	**PTEN**
**MYC***	**MYCN***	NF1	**RB1**	ROS1	**SETD2**
PAX5	**PDGFRA***	PIK3CA	SLX4	SMARCA4	SMARCB1
PIK3R1*	PTCH1	**PTEN**	SMO	TERT	**TP53**
**RB1**	RUNX1	**SETD2**	WHSC1		
SMARCA4	SMARCB1	SUFU			
SUZ12	TCF3	TET2			
**TP53**	USP7	WHSC1			
WT1					

Table S6: Target and agent pairs identified for each patient sample.

		OCCRA		OCAV3		WGS	
**Sample**	**Patient**	**Target**	**Agent**	**Target**	**Agent**	**Target**	**Agent**
1	1	0	N/A	0	N/A	N/A	N/A
2	2	0	N/A	0	N/A	N/A	N/A
3	3-Diagnosis	0	0	0	0	N/A	N/A
4	3-Relapse2	CDKN2A loss [10]	CDK4/6 inhibitors ǂ	CDKN2A loss [10]	CDK4/6 inhibitors ǂ	N/A	N/A
5	4-Relapse2	JAK1 [11]<br>CDKN2A/B loss [10]	JAK/STAT inhibitors^<br>CDK4/6 inhibitors ǂ	JAK1 [11]<br>CDKN2A/B loss [10]	JAK/STAT inhibitors^<br>CDK4/6 inhibitors ǂ	N/A	N/A
6	4-Relapse5	JAK1 [11]<br>CDKN2A/B loss[10]	JAK/STAT inhibitors^<br>CDK4/6 inhibitors ǂ	JAK1 [11]<br>CDKN2A/B loss[10]	JAK/STAT inhibitors^<br>CDK4/6 inhibitors ǂ	N/A	N/A
7	5	KRAS [12]<br>CDKN2A/B loss[10]	MEK inhibitors *<br>CDK4/6 inhibitors ǂ	KRAS [12]<br>CDKN2A/B loss [10]	MEK inhibitors *<br>CDK4/6 inhibitors ǂ	N/A	N/A
8	6-Relapse1	JAK2 amp [11]	JAK/STAT inhibitors^	0	N/A	N/A	N/A
9	7	0	N/A	0	N/A	N/A	N/A
10	8	CDKN2A/B loss[10]	CDK4/6 inhibitors ǂ	CDKN2A/B loss [10]	CDK4/6 inhibitors ǂ	N/A	N/A
11	9	0	N/A	0	N/A	N/A	N/A
12	10	CDKN2A loss [10]	CDK4/6 inhibitors ǂ	CDKN2A loss [10]	CDK4/6 inhibitors ǂ	N/A	N/A
13	11	CDKN2A/B loss[10]<br>NUP214-ABL1 [13]	CDK4/6 inhibitors ǂ<br>Tyrosine Kinase inh ^	0	N/A	N/A	N/A
14	12	CDKN2A/B loss[10]	CDK4/6 inhibitors ǂ	CDKN2A/B loss [10]	CDK4/6 inhibitors ǂ	N/A	N/A
15	13	0	N/A	0	N/A	N/A	N/A
16	14	0	N/A	0	N/A	N/A	N/A
17	15	0	N/A	MYCN amp [14]	BET inhibitors ǂ	0	N/A
18	16	CDKN2A/B loss [10]	CDK4/6 inhibitors ǂ	0	N/A	0	N/A
19	17-Relapse5	BRAF [15]	BRAF inhibitors *	BRAF [15]	BRAF inhibitors *	BRAF [15]	BRAF inhibitors *
20	18-Relapse1	KRAS amp [12]	MEK inhibitors *	KRAS amp [12]	MEK inhibitors *	KRAS amp [12]<br>HRAS amp [16]	MEK inhibitors *
21	19	TSC2 [17]<br>CDK4 amp [18]<br>MYC amp [19]	MTOR inhibitors *<br>CDK4/6 inhibitors ǂ<br>BET inhibitors ǂ	TSC2 [17]<br>CDK4 amp [18]<br>MYC amp [19]	MTOR inhibitors *<br>CDK4/6 inhibitors ǂ<br>BET inhibitors ǂ	CDK4 amp [18]<br>MYC amp [19]	CDK4/6 inhibitors ǂ<br>BET inhibitors ǂ
22	20	FGFR1 amp [20]	FGFR inhibitors *	FGFR1 amp [20]	FGFR inhibitors *	0	N/A
23	21	CDKN2A/B loss [10]	CDK4/6 inhibitors ǂ	0	N/A	CDKN2A/B loss[10]	CDK4/6 inhibitors ǂ
24	22-Relapse1	NF1 [21]<br>FGFR1 amp [20]<br>MYC amp [19]	MEK inhibitors *<br>FGFR inhibitors *<br>BET inhibitors ǂ	NF1[21]<br>FGFR1 amp [20]<br>MYC amp [19]	MEK inhibitors *<br>FGFR inhibitors *<br>BET inhibitors ǂ	NF1 loss [21]<br>FGFR1 amp [20]<br>MYC amp [19]	MEK inhibitors *<br>FGFR inhibitors *<br>BET inhibitors ǂ
25	23	0	N/A	0	N/A	0	N/A
26	24-Relapse1	NF1[21]	MEK inhibitors*	NF1[21]	MEK inhibitors*	NF1 loss [21]	MEK inhibitors*
27	25	CDKN2A/B loss [10]	CDK4/6 inhibitors ǂ	PDGFRA amp [22]	PDGFRA inhibitors*	PDGFRA amp [22]	PDGFRA inhibitors*
28	26	0	N/A	0	N/A		

* agents reviewed by the target-agent prioritization (TAP) committee and included in Pediatric MATCH trial

ǂ agents that were reviewed by the TAP committee for Pediatric MATCH and are not included in the trial

^ agents that were supported by clinical trials or case reports.

References for Supplementary Materials

1. Lih, C.J., Harrington, R.D., Sims, D.J., Harper, K.N., Bouk, C.H., Datta, V., Yau, J., Singh, R.R., Routbort, M.J., Luthra, R., et al. (2017). Analytical Validation of the Next-Generation Sequencing Assay for a Nationwide Signal-Finding Clinical Trial: Molecular Analysis for Therapy Choice Clinical Trial. The Journal of molecular diagnostics : JMD *19*, 313-327.
2. Hiemenz, M.C., Ostrow, D.G., Busse, T.M., Buckley, J., Maglinte, D.T., Bootwalla, M., Done, J., Ji, J., Raca, G., Ryutov, A., et al. (2018). OncoKids: A Comprehensive Next-Generation Sequencing Panel for Pediatric Malignancies. The Journal of molecular diagnostics : JMD.
3. Laskin, J., Jones, S., Aparicio, S., Chia, S., Ch'ng, C., Deyell, R., Eirew, P., Fok, A., Gelmon, K., Ho, C., et al. (2015). Lessons learned from the application of whole-genome analysis to the treatment of patients with advanced cancers. Cold Spring Harbor molecular case studies *1*, a000570.
4. Allen, C.E., Laetsch, T.W., Mody, R., Irwin, M.S., Lim, M.S., Adamson, P.C., Seibel, N.L., Parsons, D.W., Cho, Y.J., Janeway, K., et al. (2017). Target and Agent Prioritization for the Children's Oncology Group-National Cancer Institute Pediatric MATCH Trial. Journal of the National Cancer Institute *109*.
5. Furumoto, Y., and Gadina, M. (2013). The arrival of JAK inhibitors: advancing the treatment of immune and hematologic disorders. BioDrugs : clinical immunotherapeutics, biopharmaceuticals and gene therapy *27*, 431-438.
6. Deenik, W., Beverloo, H.B., van der Poel-van de Luytgaarde, S.C., Wattel, M.M., van Esser, J.W., Valk, P.J., and Cornelissen, J.J. (2009). Rapid complete cytogenetic remission after upfront dasatinib monotherapy in a patient with a NUP214-ABL1-positive T-cell acute lymphoblastic leukemia. Leukemia *23*, 627-629.
7. Clarke, S., O'Reilly, J., Romeo, G., and Cooney, J. (2011). NUP214-ABL1 positive T-cell acute lymphoblastic leukemia patient shows an initial favorable response to imatinib therapy post relapse. Leukemia research *35*, e131-133.
8. Grobner, S.N., Worst, B.C., Weischenfeldt, J., Buchhalter, I., Kleinheinz, K., Rudneva, V.A., Johann, P.D., Balasubramanian, G.P., Segura-Wang, M., Brabetz, S., et al. (2018). The landscape of genomic alterations across childhood cancers. Nature *555*, 321-327.
9. Ma, X., Liu, Y., Liu, Y., Alexandrov, L.B., Edmonson, M.N., Gawad, C., Zhou, X., Li, Y., Rusch, M.C., Easton, J., et al. (2018). Pan-cancer genome and transcriptome analyses of 1,699 paediatric leukaemias and solid tumours. Nature *555*, 371-376.
10. Shapiro, G.I. (2017). Genomic Biomarkers Predicting Response to Selective CDK4/6 Inhibition: Progress in an Elusive Search. Cancer cell *32*, 721-723.

11. O'Shea, J.J., Schwartz, D.M., Villarino, A.V., Gadina, M., McInnes, I.B., and Laurence, A. (2015). The JAK-STAT pathway: impact on human disease and therapeutic intervention. Annual review of medicine *66*, 311-328.
12. Janne, P.A., Shaw, A.T., Pereira, J.R., Jeannin, G., Vansteenkiste, J., Barrios, C., Franke, F.A., Grinsted, L., Zazulina, V., Smith, P., et al. (2013). Selumetinib plus docetaxel for KRAS-mutant advanced non-small-cell lung cancer: a randomised, multicentre, placebo-controlled, phase 2 study. The Lancet. Oncology *14*, 38-47.
13. Quintas-Cardama, A., Tong, W., Manshouri, T., Vega, F., Lennon, P.A., Cools, J., Gilliland, D.G., Lee, F., Cortes, J., Kantarjian, H., et al. (2008). Activity of tyrosine kinase inhibitors against human NUP214-ABL1-positive T cell malignancies. Leukemia *22*, 1117-1124.
14. Puissant, A., Frumm, S.M., Alexe, G., Bassil, C.F., Qi, J., Chanthery, Y.H., Nekritz, E.A., Zeid, R., Gustafson, W.C., Greninger, P., et al. (2013). Targeting MYCN in neuroblastoma by BET bromodomain inhibition. Cancer discovery *3*, 308-323.
15. Pratilas, C.A., Xing, F., and Solit, D.B. (2012). Targeting oncogenic BRAF in human cancer. Current topics in microbiology and immunology *355*, 83-98.
16. Kiessling, M.K., Curioni-Fontecedro, A., Samaras, P., Atrott, K., Cosin-Roger, J., Lang, S., Scharl, M., and Rogler, G. (2015). Mutant HRAS as novel target for MEK and mTOR inhibitors. Oncotarget *6*, 42183-42196.
17. Franz, D.N., Belousova, E., Sparagana, S., Bebin, E.M., Frost, M., Kuperman, R., Witt, O., Kohrman, M.H., Flamini, J.R., Wu, J.Y., et al. (2013). Efficacy and safety of everolimus for subependymal giant cell astrocytomas associated with tuberous sclerosis complex (EXIST-1): a multicentre, randomised, placebo-controlled phase 3 trial. Lancet *381*, 125-132.
18. Rader, J., Russell, M.R., Hart, L.S., Nakazawa, M.S., Belcastro, L.T., Martinez, D., Li, Y., Carpenter, E.L., Attiyeh, E.F., Diskin, S.J., et al. (2013). Dual CDK4/CDK6 inhibition induces cell-cycle arrest and senescence in neuroblastoma. Clinical cancer research : an official journal of the American Association for Cancer Research *19*, 6173-6182.
19. Bandopadhayay, P., Bergthold, G., Nguyen, B., Schubert, S., Gholamin, S., Tang, Y., Bolin, S., Schumacher, S.E., Zeid, R., Masoud, S., et al. (2014). BET bromodomain inhibition of MYC-amplified medulloblastoma. Clinical cancer research : an official journal of the American Association for Cancer Research *20*, 912-925.
20. Andre, F., Bachelot, T., Campone, M., Dalenc, F., Perez-Garcia, J.M., Hurvitz, S.A., Turner, N., Rugo, H., Smith, J.W., Deudon, S., et al. (2013). Targeting FGFR with dovitinib (TKI258): preclinical and clinical data in breast cancer. Clinical cancer research : an official journal of the American Association for Cancer Research *19*, 3693-3702.

21. See, W.L., Tan, I.L., Mukherjee, J., Nicolaides, T., and Pieper, R.O. (2012). Sensitivity of glioblastomas to clinically available MEK inhibitors is defined by neurofibromin 1 deficiency. Cancer research *72*, 3350-3359.

22. Dai, X., Theobard, R., Cheng, H., Xing, M., and Zhang, J. (2018). Fusion genes: A promising tool combating against cancer. Biochimica et biophysica acta. Reviews on cancer *1869*, 149-160.
